# Upcycling Spent Coffee Grounds: Approaches, Emerging Concepts and Applications

**DOI:** 10.3390/foods15122155

**Published:** 2026-06-15

**Authors:** Sreehitha Pilli, Jeyan Arthur Moses, Senthilkumar Thiruppathi, Sinija Vadakkepulppara Ramachandran Nair, Loganathan Manickam

**Affiliations:** 1 Department of Food Process Engineering, National Institute of Food Technology, Entrepreneurship and Management, Thanjavur, MoFPI, Government of India, Thanjavur 613005, Tamil Nadu, India; 2 Faculty of Sustainable Design Engineering, University of Prince Edward Island, 550 University Avenue, Charlottetown, PE C1A 4P3, Canada; 3 Department of Food Packaging and Storage Technology, National Institute of Food Technology, Entrepreneurship and Management, Thanjavur, MoFPI, Government of India, Thanjavur 613005, Tamil Nadu, India

**Keywords:** spent coffee grounds, waste valorization, bioactive compounds, extraction techniques, circular economy

## Abstract

Spent coffee grounds (SCG) are generated in millions of tonnes annually due to rising global coffee consumption, posing significant challenges, including greenhouse gas emissions, waste-disposal problems, and the loss of valuable compounds like caffeine, dietary fibre, phenolics, antioxidants, proteins, and lipids, offering prospects for potential valorization. Its composition is influenced by several factors. This review focuses on recent advancements in the valorization of SCG across sectors such as food, nutraceuticals, bioenergy, and packaging. The emphasis is on pretreatment, extraction, and bioconversion methods, as well as current research gaps, limitations, and future directions. SCG valorization is oriented toward integrated, multi-product biorefinery systems based on green extraction and bioconversion technologies to recover high-value compounds in both the food and non-food sectors. Nonetheless, industrial scalability is limited by composition variability, energy-intensive processing, techno-economic constraints, and safety and regulatory issues that remain unresolved. The shortcomings, such as inadequate standardized characterization, toxicological validation, and pilot-scale studies, are critical gaps. Scalable, energy-efficient processes, AI-assisted optimization, and regulatory alignment development should be a priority in future research, so that sustainable and commercial deployment is possible.

## 1. Introduction

**Figure 1:**
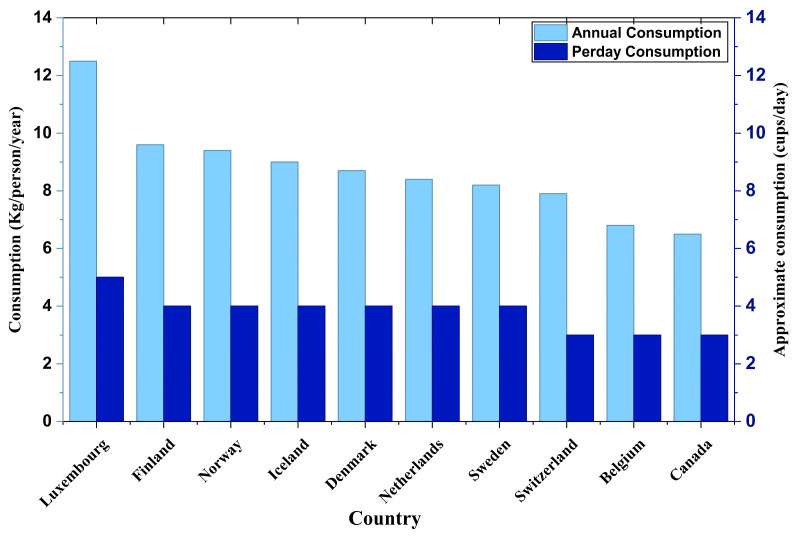
Per capita coffee consumption of the top 10 countries in 2025 (Adapted from [6]).

Coffee is the most consumed beverage worldwide and a highly valuable product. After oil and its products, coffee is the second-largest traded commodity, with wide varieties including arabica (*Coffea arabica*) and robusta (*Coffea canephora*), which accounted for 62.7% and 37.3% of exports in 2023, respectively [1]. Global coffee production reached approximately 175.32 million 60 kg bags (10.52 million tonnes) in the year 2024–2025 and has continued to increase, with the recent data representing production of 178.85 million 60 kg bags (10.73 million tonnes) in 2025–2026, reflecting a steady annual growth of about 2% [2]. Correspondingly, global consumption has reached approximately 180.2 million 60 kg bags, indicating growing demand for coffee [3], with notably high per capita intake in major consuming countries (Figure 1). The rapid rise in global consumption, with an estimated 2.3 billion cups of coffee consumed every day, has significantly increased the amount of spent coffee grounds (SCG), with an estimated annual production of 6 million tonnes [4]. For every tonne of raw coffee beans utilized, approximately 650 kg of SCG is generated. Every 30 g of coffee powder used in a double-shot espresso produces 45–60 g of SCG, an increase in SCG weight relative to the ground coffee powder due to hydration [5].

During coffee production, various operations are involved, including harvesting, pulping of cherries, fermentation, and hulling in wet processing, as well as roasting and brewing. Each operation generates by-products at different stages, making coffee production one of the most prolific sources of agro-industrial residues in the world. Although organic in nature, SCG are generally rejected as waste, and almost half of coffee-processing residues are deposited in landfills, which leads to the accumulation of solid waste and the related burden on the environment [5]. Some of the environmental issues that improper disposal of SCG may bring about include uncontrolled decomposition, generation of leachates, and emission of greenhouse gases, which include methane and carbon dioxide, when under landfill conditions. Moreover, SCG has traces of bioactive substances like caffeine and polyphenols that can be toxic to soil and water when discharged in uncontrolled wastewater [7]. These features underscore SCG as not only a disposal problem but also an organic waste that could be a source of pollution, unless well handled. Thus, the focus has been shifted to sustainable valorization strategies transforming SCG into value-added products, which will aid in waste reduction and will be a part of the approach to a circular economy that will reduce the impact on the environment [8].

**Figure 2:**
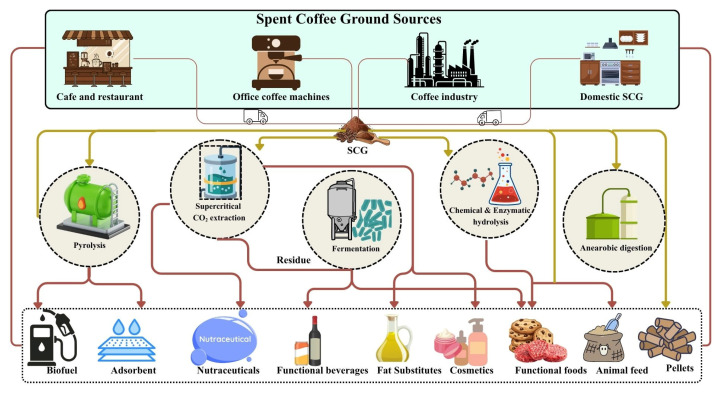
Comprehensive biorefinery framework for SCG valorization.

SCG has a unique biochemical composition with high polysaccharides, lipids, fibre, protein, and bioactive compounds, especially caffeine, chlorogenic acids, and other polyphenols that have many biological attributes, including potent antioxidant, anticarcinogenic, anti-allergic, anti-inflammatory, antimicrobial, and antitumor properties. As well SCG having beneficial properties related to neuroprotection and residual nutrients, which enable reutilization in food and non-food applications through sustainable bioprocess frameworks [8]. These phytoconstituents could be used in the development of nutraceuticals and functional foods, and SCG’s high lignocellulose and carbon content hold potential for bioenergy production. There are numerous pathways for valorizing SCG to produce low-cost bioenergy or high-value compounds, as illustrated in Figure 2 [9].

Recent studies have explored diverse valorization techniques to integrate SCG into numerous applications across the food and non-food sectors. The food-related valorization of SCG has focused on its potential as a constituent of functional foods, nutraceuticals, fat substitutes, fermented foods, biodegradable packaging materials, and other applications in food [10]. In the non-food sector, SCG utilization is focused on supplements for animal feed, cosmetics, biopolymers, biocatalysts, soil amendment, and other bioenergy applications [11,12]. Although various valorization pathways have been developed, it remains fragmented due to its challenges, such as variability in composition, scalability constraints, standardization issues, and safety concerns, including microbial contamination, heavy metal risks, and regulatory requirements, making it inadequate for use mainly in food applications [13].

The existing reviews on SCG valorization lacked integration, with most reviews being specific to either food or non-food applications, providing limited coverage of integrated valorization approaches. Furthermore, recent developments in the valorization of SCG have not been consolidated into a unified framework that integrates various approaches to valorization. The present review aims to provide an overview of recent advancements in the valorization of SCG across food and non-food categories, which provides a detailed framework about the food applications with advanced valorizing strategies. In addition, this review highlights the concepts of safety, circular economy, emerging trends, and future directions through integrated approaches.

## 2. Nutritional and Functional Attributes of SCG

### 2.1. Composition of SCG

The SCG contains polysaccharides, lipids, proteins, minerals, and bioactive compounds in significant amounts. The dried SCG (dry weight basis) contains carbohydrates 40–50%, lipids 20–25%, proteins 10–15%, and moisture 10–5% [14]. A comprehensive overview of the compositional profile and key constituents of SCG is presented in Table 1.

However, this composition may be affected by many factors, including coffee bean variety, geographical location, cultivation method, harvesting stage, processing techniques, and brewing method. Nonetheless, the variation in SCG composition is within the limit [15]. SCG contains a high portion of polysaccharides (45–46%), primarily composed of cellulose (10–20%) and hemicellulose (39–40%). The hemi cellulosic fraction is mainly constituted of galactomannans, with mannose (21–46%) and galactose (15–32%). The insoluble and soluble dietary fibre content is around 35% and 8%, respectively. Lignin is an unstructured, heterogeneous polymer with a lower concentration in SCG compared to other biomasses. Its content varies from 22.2 to 33.6%, composed of phenolic compounds such as coniferyl, p-coumaryl alcohol, and sinapyl, chemically linked through various linkages [14]. The lipids in the range of 18–20%, with the predominant fatty acids being linoleic acid (36–42%), palmitic acid (32%), oleic acid (10–12%), and stearic acid (7–10%), which have potential in the cosmetic industry due to their antioxidant and anti-inflammatory properties [16]. It also contains comparatively high protein (13–17.54%). SCG has proven to be a rich source of bioactive compounds, including phenolics and flavonoids. The major phenolic compounds were chlorogenic acids, caffeic acids, gallic, ferulic, and protocatechuic acids, and in minor amounts, tannins, quercetin, catechin, and kaempferol are also present. The concentration of these phenolic compounds is 16–173.3 mg gallic acid equivalents/g of SCG. These compounds bind to carbohydrates and other macromolecules, which have antioxidant, anti-inflammatory, anti-cancer, anti-hypertensive, anti-adipogenic and anti-diabetic properties; all these properties make it more promising. The recovery of bioactive compounds depends on many factors, including the extraction method, process conditions, source of SCG, and other factors [17]. At last, SCG also has mineral content, in which the potassium content is more around 11,700 mg/kg of SCG, and other microelements are calcium (1200 mg/kg), phosphorous (1800 mg/kg), sulphur (1600 mg/kg), magnesium (1900 mg/kg); in minor portions, copper, cobalt, manganese, zinc and iron were present around 19, 15, 29, 8, and 52 mg/kg, respectively, making it promising for the application of SCG as fertilizer to improve soil fertility [18]. Nevertheless, in food systems, these minerals may offer some nutritional value when added in controlled amounts, so long as safety issues, including the contamination of heavy metals and bioavailability, are properly resolved.

### 2.2. Factors Affecting the Composition of SCG

The composition of SCG is greatly affected by many parameters, including coffee variety, geographical origin (soil, climate, and altitude variations), brewing method, and bean processing conditions, such as roasting temperature and grind size (Table 2).

A recent study reported that coffee variety and brewing cycles affected the bioactive compounds of SCG. They studied three levels of brewing cycles at 92–95 °C temperature and 900 kPa pressure on dried SCG samples from the arabica and robusta coffee varieties. The total phenolic content (TPC), total flavonoid content (TFC), and caffeine concentrations were varied in the ranges of 6.80 to 31.31 mg GAE/g DW, 6.2 to 9.1 mg QE/g DW, and 14 to 55 mg/g, respectively [22]. Similarly, brewing methods and the degree of coffee bean roasting have been shown to affect the composition of SCG profoundly. Notably, brewing using water at high temperatures and a medium roast degree has been shown to result in the retention of caffeoylquinic acids. The sample also retained antioxidant activity compared to cold brew and espresso methods, although dark roast status resulted in a reduction in chlorogenic acid [23]. Andrade et al. (2022) studied the effect of geographical location on the SCG phenolic and antioxidant activity by collecting SCG from different geographical locations (Guatemala, Colombia, Brazil, Timor, and Ethiopia) and concluded that there is a correlation between the bioactives and geographical origin [24].

### 2.3. Health Benefits

SCG contains high-value bioactive compounds, including chlorogenic acid, caffeic acid, trigonelline, and melanoidin, which have many health benefits. It has been reported that chlorogenic acids improve blood pressure control, anti-adipogenic action, glucose metabolism, and antioxidant capacity [16]. Caffeine is also recognized for its neuroprotective properties, memory enhancement, and cognitive function improvement. Another well-known compound is trigonelline, which is a neuroprotector with the ability to control oxidative stress, glucose, lipid, and brain activity. At last, melanoidins as a functional food additive demonstrated strong antioxidant, antibacterial, and gastrointestinal health-modulating properties [5]. Although SCG has a high level of bioactive compounds, their biological activities following digestion will rely on bioaccessibility and bioavailability. Several in vitro gastrointestinal digestion and Caco-2 cells studies showed that chlorogenic acids, caffeoylquinic acids, melanoidins, flavonoids and condensed tannins are only partially stable during gastrointestinal digestion and bioaccessible in the gastrointestinal tract [25]. The release of condensed tannins and bound phenolics from the fermentation of the colon caused a 1–8-fold increase in antioxidant activity, as reported by [26]. In a similar study, Monente et al. (2015) also reported that caffeoylquinic acid and feruloylquinic acid were relatively stable during digestion, but systemic bioavailability was only about 1% of the total chlorogenic acids, which cross the Caco-2 intestinal barrier [25]. The antioxidant activity, the phenolic bioaccessibility, and the α-glucosidase inhibitory activity of bakery products enriched with SCG were also observed to increase after simulated digestion [26]. Animal studies also showed that there was a significant reduction in hepatic lipid accumulation and an increase in lipid excretion without acute toxicity after SCG supplementation [27]. However, most of this evidence of the health benefits of SCG bioactives is restricted to in vitro and animal studies, and there is a lack of well-designed clinical studies in humans.

## 3. Valorization of SCG: Pretreatment Approaches

Pretreatment techniques are employed at the preliminary processing stage in the valorization of SCG, with the main objective of modifying its morphological and structural properties to enhance process efficiency. These methods improve the accessibility of cellulose and hemicellulose for subsequent hydrolysis and conversion processes. A comparative overview of different pretreatment strategies and their efficiencies is presented in Table 3. The typical aims of pretreated processes must be as follows: 1. SCG hemicellulose fraction degradation; 2. reduction of the formation of any inhibitors or by-products for further processes; 3. removal of lignin content; 4. production of highly digestible pretreated SCG.

### 3.1. Physical Pretreatment Techniques

Physical pretreatment techniques play a significant role in improving mass transfer and structural disruption, thereby enhancing both extraction and conversion efficiency. Microwave-assisted pretreatment may be widely used for SCG due to its rapid heating and effective biomass disruption. SCG can be subjected to a high-power microwave digester with a 1000 W capacity and 140-170 °C for 10 min. This will significantly enhance biomass deconstruction, which in turn facilitates enzymatic hydrolysis or fermentation [35]. Similarly, hydrothermal pretreatment has been reported as an effective method to improve the yields of phenolics and hydrochars by significantly altering the lignocellulosic structure of SCG. Treatment has been carried out at 200–260 °C for 1–3 h, leading to partial delignification and the generation of hydrochars, with conditions at 200 °C for 1 h, preserving higher amounts of phenolic compounds in the solids [36]. The extreme conditions of pretreatment lead to the gradual degradation of phenolics and decrease their recoverability in downstream processes.

In the application of hydrodynamic cavitation (HC) as pretreatment for the SCG, the biodegradability was improved. In this process, an orifice plate with a conical concentric hole with 3 mm inlet and 10 mm outlet diameter was operated at 5 bar pressure for a 20- or 30-min duration. This enhanced the biodegradability by dissolving the organic carbon content [37]. A novel non-thermal physical pretreatment for enhancing the recovery of bioactive substances from the SCG is cold atmospheric plasma (CAP). It generates reactive species with plasma, which in turn causes surface-level structural and physicochemical changes that improve solvent penetration. In this approach, a 1 mm thick SCG layer was placed at a distance of 16 mm from the electrode, and the treatment was carried out for 15 min. The pretreated sample, subjected to ultrasound-assisted extraction, showed a significant increase in the yields of bioactive compounds [38]. To improve the oil yields from the SCG, Leal Vieira Cubas et al. (2020) used non-thermal plasma as a pretreatment, which increased yields by up to 30% and also escalated antioxidant capacity, possessing anti-inflammatory and immunomodulatory agents [39].

### 3.2. Physiochemical Pretreatments

The physicochemical pretreatments significantly affect the polyphenol content in SCG. This further affects lignocellulolytic enzyme production in downstream applications. The organic solvents and sequential EDTA, alkaline (NaOH or KOH)-based pretreatments significantly affected polyphenol content by reducing it compared to untreated SCG. Untreated SCG contained higher amounts of polyphenols, around 32 mg GAE/g DW of SCG. Higher cellulase (1.33 ± 0.06 IU/mL) and pectinase (0.32 ± 0.02 IU/mL) activities were also observed, showing a positive correlation with polyphenol content. Conversely, xylanase and peroxidase activity attained higher levels after boiling water and 1% EDTA treatment, which were 0.31 ± 0.002 IU/mL and 15.56 ± 0.56 IU/mL, respectively [39]. An effective and gentle physicochemical pretreatment for fractionating the biomass is ultrasound-assisted alkaline potassium permanganate treatment. The method enabled significant delignification while preserving the polysaccharide fraction due to the strong oxidative selectivity of KMnO_4_ towards lignin and the cavitation effect induced by ultrasonication. High cellulose recovery of 98% and moderate lignin removal with 46% were attained under ideal conditions with 4% *w*/*v* KMnO_4_ and 20 min ultrasonication at room temperature, leading to a 1.7-fold increase in reducing sugar yields after enzymatic hydrolysis.

### 3.3. Chemical Pretreatments

Chemical pretreatment enables the substantial extraction of high-value compounds, including oligosaccharides, fermentable sugars, and cellulose-rich materials, for downstream applications. The sulfuric acid pretreatment is effective for oligosaccharide (OS) extraction from SCG. Yields of 21.4–22.4 g per 100 g of SCG were obtained under circumstances of a solid-to-liquid ratio of 1:40, 1.43% *v*/*v* H_2_SO_4_ at 168.6 °C for 20 min. The process also enabled the recovery of mannobiose-type oligomers with a 1:1:1 mannose:galactose:arabinose ratio, indicating its efficiency [17]. Conversely, sodium hydroxide pretreatment was reported as a promising method for the extraction of polysaccharides from the SCG. Treatment with 4 M sodium hydroxide solution at 25 °C recovered 70% polysaccharides with higher antioxidant activity. The extracted polysaccharides have the capacity to inhibit the growth of fungi by 41.3 to 54.6% [40]. In parallel, the organoslov pretreatments, including ethanol and formic acid–acetic acid–water, decreased the lignin content to 7.1 from 15.5% in untreated SCG and increased cellulose content from 11.5 to 29.5% on a dry basis. The significant delignification achieved through pretreatment allowed for efficient peracetic acid pulping, confirming that selective lignin removal is a key step towards generating cellulose-rich fractions from SCG. These fractionated streams are further treated with sequential alkali treatment and NaOH/H_2_O_2_ to increase the enzymatic accessibility through oxidative delignification and hemicellulosic chain removal. The treatment increased the total sugars from 37.5% to 74.1%. The resulting sugars were highly enriched in mannose at 45.2% and glucose at 2.2% [41]. The dual hydrolysis procedure involving diluted acid hydrolysis and cellulase treatment was adopted to increase reducing sugars, including galactose, glucose, and arabinose, for lactic acid production with a yield up to 98%.

## 4. Upcycling and Repurposing SCG: Advances in Technologies

Upcycling SCG has gained significant attention as a sustainable practice for the valorization of agro-industrial waste to produce high-value products. The emergence of biological, chemical, and emerging non-thermal and novel thermal techniques for the efficient extraction of bioactive compounds, functional products, and fuel from SCG has improved resource utilization and the circular economy. The circular economy involves integrating sustainable, eco-friendly, and cost-effective approaches.

### 4.1. Biological Techniques

The process of turning SCG into value-added products using microorganisms or enzymes in mild, eco-friendly settings is known as biological valorization. These techniques make use of SCG’s rich lignocellulosic and nutritional composition as substrates for enzyme synthesis, microbial growth and biotransformation.

#### 4.1.1. Fermentation

Fermentation enables the conversion of SCG into valuable compounds, including organic acids, such as lactic acid, citric acid and acetic acid, which play an important role in food industry applications. The high cost of conventional fermentation substrate is the major challenge; SCG can be used as an alternative substrate with pretreatment to produce lactic acid with a productivity of 0.95 g/L/h within 24 h. These findings strengthen SCG as a good substrate for lactic acid production through fermentation [42]. Furthermore, a cascade of applications through fermentation enables SCG to be converted into valuable compounds, including fermentable sugars, volatile fatty acids (VFAs), yeast-based single-cell protein, and biofuels. The VFAs extracted from SCG through fermentation can be used to cultivate the yeast cells of *Candida maltosa*. This process has been reported to produce single-cell proteins with a protein content of up to 43.7%, corresponding to a concentration of 4.6 g/L, as reported by [43]. As well, the biomass derived from fermentation can be used as a biosorbent for textile dye removal [8]. This technique can be used for enzyme production and wastewater treatment, which can be supported by circular bioeconomy strategies.

In addition to submerged fermentation, solid-state fermentation (SSF) is another emerging application to convert SCG into useful metabolites. The fungal strains *Aspergillus oryzae* and *Aspergillus awamori* cultivated through SSF increase the growth of strains, leading to an enhancement of the oligosaccharide yields up to 14.15 mg/100 g, as well as reducing browning by degrading melanoidins [44]. These oligosaccharides showed prebiotic activity in gut health, indicating that the SSF of SCG could provide a potential prebiotic component. There is a potential for developing fermentation-based valorization towards a functional food. The SCG as substrate under SSF showed good results in the production of laccase with 14.62 U/g of laccase activity [8]. The new key strategy in valorizing SCG through fermentation is selective fermentation. This is a commercially viable technique for large-scale production, and it improves the economic feasibility by selectively fermenting the target molecules and reducing the production of by-products [45].

#### 4.1.2. Enzymatic Hydrolysis

One of the most effective methods for hydrolyzing complex SCG structures into simple bioavailable compounds without the creation of inhibitors is enzymatic hydrolysis, a low-energy and environmentally friendly process. Following partial delignification via sequential pretreatments, the enzymatic hydrolysis of SCG demonstrated considerable potential in the conversion of carbohydrates to fermentable sugars. Jin et al. (2020) successfully optimized enzymatic hydrolysis conditions for SCG and reported maximum sugar production at pH 4.8, 55 °C and a low cellulase dosage of 0.01 mL, and the results showed that the sugar concentration was 1.824 mg/mL [46]. In another study, β-mannanase was used in combination with roasting pretreatment for the valorization of SCG through enzymatic hydrolysis, showing a pronounced improvement in the sugar yields up to 17.43%. The TPC concentration also increased by 6.39-fold, which was 291.86 GA/g of SCG [47]. The combined action of cellulase and mannanase significantly increased the sugar yields, with high glucose levels of 15.3 mg/mL and mannose levels of 10.6 mg/mL, which represent efficient saccharification of both cellulose and galactomannan fractions [41].

### 4.2. Chemical Techniques

#### 4.2.1. Acid Hydrolysis

Acid hydrolysis is effective for depolymerizing cellulose and hemicellulose to produce fermentable sugars. Through acid hydrolysis, oligosaccharides can be extracted from SCG at 200 °C in a closed reactor setup, exhibiting functional properties. The extracted oligosaccharides, with high potential for prebiotic activity, created an innovative pathway for functional food formulations. Another study reported that a 2% sulfuric acid concentration recovered oligosaccharide yields, which varied from 1.65 to 22.40 g/100 g of dry SCG, with the variation due to process conditions during coffee brewing [17]. Instead of using acid hydrolysis as a direct valorization technique, this treatment can be used as a pretreatment. This enables the produced hydrolysates to be used for further downstream applications, which have great potential for the recovery of oligosaccharides with high prebiotic activity.

### 4.3. Non-Thermal/Novel Thermal Techniques

#### 4.3.1. Supercritical CO_2_ Extraction

Supercritical CO_2_ (SC-CO_2_) extraction is a sustainable, non-thermal technique for the valorization of SCG. This is mainly for the extraction of bioactive and lipid compounds, which have great potential in cosmetic and food industry applications. Recent studies have shown that the high moisture content of SCG, around 50%, which requires energy-intensive drying, can instead be used as a cosolvent in the extraction process [48]. For the extraction of bioactive and lipid compounds, the optimized process parameters, determined using response surface methodology, were 265 bar and 55 °C. The moisture content of SCG plays a crucial role in the extraction of polar molecules by disrupting the caffeine and chlorogenic acid interactions within the SCG matrix.

The studies provide evidence of the efficacy of SC-CO_2_ extraction for the valorization of SCG, especially for coffee oil extraction. Under optimized conditions, it was found to be comparable to conventional Soxhlet extraction in terms of oil yield and extraction time [48]. The use of cosolvents, including isopropanol, ethanol, and ethyl lactate, alongside the solvent, has been reported to improve extraction efficiency. These cosolvents reduce the extraction time by half compared with pure CO_2_ as the solvent and increase the antioxidant capacity of the extracted oil. However, no significant effect was observed on the principal compounds, including the palmitic and linoleic esters [49]. In the extraction of bioactive compounds, SC-CO_2_ has shown significant results in the sequential extraction process. The sequential extraction process enhanced the yields of fatty acid profile, TPC, and antioxidant activity under the processed conditions (80 °C, 200 bar, 2 mL/min), which were 21.54 ± 0.56 mg GAE/g DW TPC with 4196 ± 140 µmol TE/100 g AA, respectively [48]. Romano et al. (2023) reported that extractions of bioactive compounds using SC-CO_2_ with 5% ethanol for 1 h provided the highest yields with high total polyphenolic contents and antioxidant activity compared to extraction with hexane for 5 h [50]. In addition, in bioactive compound applications, SC-CO_2_ extraction has been explored as a crucial upstream processing step in biorefinery concepts for material-focused valorization of SCG. The extracted oil was found to be suitable as a carbon source to produce polyhydroxyalkanoates, thus emphasizing the compatibility of lipids obtained from SC-CO_2_ extraction with further valorization processes.

#### 4.3.2. Pressurized Liquid Extraction

The most effective technique for extracting oil from the SCG is pressurized liquid extraction (PLE), and it is now being widely used for the extraction of bioactive compounds as well. It is mainly pressure- and temperature-dependent, typically between 50 and 200 °C and 35 to 200 bar, respectively. Compared with conventional solid–liquid extraction, supercritical fluid extraction (SFE), and SFE + cosolvent systems, PLE demonstrated superior extraction efficiency, with relative recovery rates ranging from 92.02% to 127.5% [51]. Recent studies demonstrated the efficiency of PLE in the recovery of caffeine and polyphenolic compounds from SCG. Optimized conditions (48% ethanol, 160 °C, 25 min, 1700 psi) yielded the highest number of total polyphenols and caffeine. The extraction resulted in 15.99 mg gallic acid equivalents/g DW and caffeine 1.15 mg/g dry weight, with the antioxidant activity of 101.8 µmol ascorbic acid equivalents/g DW [52]. Through a sequential extraction process, it recovered maximum phenolic compounds with great antioxidant activity under optimized conditions. Toda et al. (2021) studied the extraction of SCG oil with PLE using ethanol and water [51]. They reported that the hydration of the solvent negatively affected the recovery of oil from the SCG, but the increase in temperature had a more pronounced effect on the recovery. The maximum recovery obtained with ethanol was around 92.02%, which was reduced to 83.87% with the diluted solvent (ethanol + H_2_O) under the same conditions of pressure and temperature. All these studies demonstrate the efficiency of PLE in the valorization of SCG into high-value functional compounds, thereby confirming its potential for sustainable use across various industries.

#### 4.3.3. Ultrasound-Assisted Extraction

Ultrasound-assisted extraction (UAE) is a non-conventional method that uses high-frequency ultrasound waves, around 20 kHz, to induce cavitation within the extraction medium. The cavitation will cause the sudden rupture of the cell wall of the complex cellulosic matrix of the SCG, leading to the leaching of the intercellular components, including the lipids and bioactive components, and an increase in surface area for more solvent absorption. Due to thermal and non-thermal acoustic effects, it also causes the physical and chemical modifications in the lignocellulosic matrix of the SCG, which may affect the nutritional and functional properties [53]. A recent study has demonstrated the efficiency of the single-stage ultrasound-assisted aqueous extraction for the valorization of the SCG. The process enabled the recovery of bioactive compounds in the liquid phase, including caffeine, polyphenols, and the melanoidins, which were 400.1, 800.4, and 2100.2 mg/100 g, respectively. Simultaneously, the solid phase contains high dietary fibre, which can be utilized in functional foods, which was representing the recovery of multiple value-added compounds using water as a green solvent [54].

Osorio Arias et al. (2023) demonstrated that the highest concentration of chlorogenic acid, 85 ± 0.6 mg/kg FW, was extracted with the UAE at 60% amplitude for 15 min, which represents the most convenient and eco-friendly method for the extraction of bioactive compounds [55]. UAE can also be used for the extraction of oil from SCG under optimized process conditions at 50 to 60 °C and an L/S ratio of around 16 mL/g of SCG, which will result in high yields. In addition, combining the UAE with hydrothermal delignification as pretreatment leads to the highest concentration of phenolic compounds. Many other studies showed that the UAE is a significant method for the sustainable valorization of SCG by converting it into high-value products, which can be incorporated into the development of functional foods, nutraceuticals and many other downstream applications [29].

#### 4.3.4. Microwave-Assisted Extraction

Microwave-assisted extraction (MAE) is one of the most promising techniques for extracting valuable compounds from SCG with low extraction time and higher yields. The microwaves will propagate into the SCG matrix, dissolving the target component into the solvent. Common solvents for SCG using the MAE technique are ethanol, water, methanol, and acetone, but ethanol is preferred for nutraceutical purposes [56]. The MAE, in combination with green solvents, including ethanol and water, has been proven to be a significant extraction method for the polyphenols from the SCG. As the ethanol percentage in the solvent mixture decreased, yields decreased, but it positively affected the antioxidant effect. The yields of polyphenols recovered from the SCG through the MAE are in the range of 31.79 ± 0.25 mg GAE/g DW to 117.7 ± 6.1 mg GAE/g DW; all these variations in the yields of phenolic compounds are due to the extraction conditions and coffee processing conditions [28]. Using the MAE, the most abundant bioactive compound from SCG was chlorogenic acid, recovered at around 84 ±  2.8 mg/g, which has numerous health benefits, including antioxidant, antimicrobial, and anti-inflammatory properties [56]. Not only for the extraction of bioactive compounds, but it can also be used for the generation of absorbent material from SCG to treat the wastewater. It can also produce effective activated carbon, which reduces the organic compounds and chemical oxygen demand within 6 h [56]. Overall, the studies stated that the MAE is an effective method for the recovery of the bioactive compounds within a short duration from the SCG, which leads to sustainable valorization and creates a circular economy.

#### 4.3.5. Subcritical Water Extraction

Subcritical water extraction (SWE) is a novel, eco-friendly, and sustainable extraction technique that uses water as a solvent at pressures and temperatures below its critical point Tc = 374.15 °C, Pc = 22.1 MPa. To recover carbohydrates, polyphenols, and the oil from SCG, the most effective method is SWE. It will target all components and extract them together as a green solvent [29]. In a comparative study of solid–liquid extraction (SLE) and SWE for the recovery of phenolic compounds from SCG, the SWE at 150 °C showed a significant effect on the recovery rate of 331.61 ± 27.85 mg GAE/g dr of TPC with higher antioxidant activity [57]. Recent studies demonstrated that SWE in combination with high-pressure carbon dioxide has enhanced the recovery of phenolic compounds from coffee by-products, which include SCG under optimal conditions (189 °C, S/L ratio of 0.024 g/mL, 54 min) [58]. J. W. Lee et al. (2025) reported that the temperature of SWE affects so many parameters, including antioxidant activity, phenolic compound recovery, prebiotic activity, and protein recovery [59]. The integrated approach of SWE and hydrothermal carbonization for SCG was effective in the development of protein isolates, resulting in an improved yield from 16.9 (control) to 33%. It also produced superior quality hydrochar with low nitrogen content around 0.23%.

However, a recent study on SWE found that SCG treatment at 250 °C was effective in reducing the solids by solubilizing the biomass. It also produced antimicrobials, including organic acids and 5- hydroxymethylfurfural. At the elevated temperature, the Maillard reactions inhibit protein recovery, while temperatures under 200 °C promote prebiotic activity for *Lactobacillus rhamnosus GG* and *Enterococcus faecium* [29]. These studies highlighted the significant potential of SWE in the valorization of SCG to produce commercially viable products across the value chain.

#### 4.3.6. Natural Deep Eutectic Solvent Extraction

To extract high-value compounds from the SCG, natural deep eutectic solvent extraction (NADES), a green extraction method, uses green solvents that are composed of hydrogen bond donors (HBD) or acceptors (HBA) such as organic acids, polyalcohols, sugars, and amino acids. These solvents will combine with betaine, choline chloride, or with combinations of organic acids and sugars or polyalcohols and amino acids [60]. As SCG are rich in phenolic compounds, the extraction was performed using a NADES prepared from citric acid:mannitol (1:1) and water (10–50%) at 40–80 °C for 2 h. This yielded up to 1620.71 ± 3.72 mg GAE/L, which proves it to be one of the best replacements for conventional solvents to recover phenolic compounds [60]. Recent studies demonstrated that microwave and ultrasound-assisted NADES-SCG extraction showed efficient extraction of bioactive compounds using betaine-glycerol and choline chloride-glycerol DES. Also, the residue was successfully converted into valuable compounds using a fermentative process for butanol production with 7.1 g/L [61]. There are multiple studies on the extraction of phenolic compounds from SCG using NADES with different DES. These include betaine:triethylene glycol (1:2), choline chloride:lactic acid (1:9) and proline:glycerol:water (2:5:11.5). The recovery ranged from 15.86 ± 0.10 mg GAE/g DW to 1038.9 ± 100.4 mg GAE/L; the recovery variation was due to the percentage of water and the molar ratios of DES [62]. These studies showed that NADES is a sustainable and efficient alternative to traditional solvent extraction methods for producing SCG extracts with various applications in functional food, cosmetics, and nutraceuticals.

#### 4.3.7. Supramolecular Extraction

Supramolecular solvents or SUPRAS are nanostructured liquids formed by the self-assembly of amphiphilic molecules. Thus, they allow a green, efficient, alternative solvent technology to extract bioactive compounds, providing an opportunity for the valorization of SCG through an emerging technology [63]. Due to its higher extraction efficiency, it can be used to extract caffeine, chlorogenic acids, and caffeic acids from the SCG, as reported by [64]. Through supramolecular extraction using 47.60% water, 27.30% 1-hexanol, and 25% ethanol, 3.50 mg/g, 3.90 mg/g, and 0.40 mg/g of caffeine, 5-CQA, and caffeic acid were extracted [64]. Additionally, water-1-propanol mixtures of monolaurin-based bioSUPRAS prepared by sulphate-induced self-assembly showed higher yields in the simultaneous extraction of polyphenols and carotenoids from SCG at room temperature for 15 min. The extractant system exhibited very high selectivity, with monolaurin loading exceeding 98%, indicating greater potential for SCG valorization. The bioactives extracted using SUPRAS, composed of decanoic acid and hexanol as amphiphiles, with water–ethanol or water–tetrahydrofuran as hydro-organic media for a 1 min extraction time from SCG, exhibited antimicrobial activity against S. aureus and B. cereus [64].

### 4.4. Advantages and Limitations of the Various Valorization Approaches

Advanced valorization systems for SCG vary considerably regarding efficiency, sustainability, cost, and industrial applicability. Biological methods like fermentation and enzymatic hydrolysis are eco-friendly and function under mild settings; nonetheless, they require longer durations for processing [65]. Chemical methods include acid hydrolysis, enabling swift depolymerization and enhanced extraction efficiency. But due to the use of caustic substances, there is a potential for the generation of inhibitory compounds [66]. Conversely, novel non-thermal and eco-friendly techniques such as SC-CO_2_, SWE, PLE, MAE, UAE, NADES, and SUPRAS extraction provide superior selectivity, less solvent utilization, and increased recovery rates. Nonetheless, substantial operational expenses, energy demands, and scalability obstacles persist as significant constraints for extensive industrial deployment [67].

## 5. Food and Non-Food Applications of SCG

SCG has attracted significant attention due to its versatility in the food and non-food sectors. An overview of applications of SCG is presented in Table 4.

### 5.1. Food Applications

**Figure 3:**
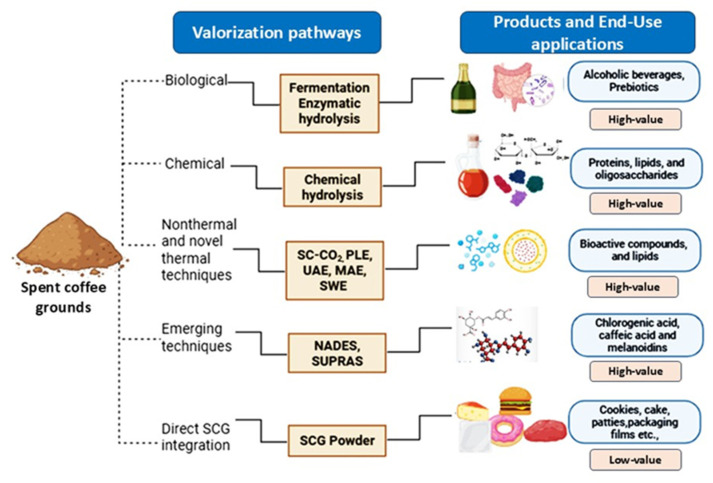
Integrated valorization pathways of SCG for food applications. Credits: Biorender.com.

SCG is rich in polysaccharides and bioactive compounds with greater antioxidant, anti-inflammatory, and antibacterial activity, as well as many health benefits, and has potential in many food applications, including nutraceuticals, functional foods, beverages, and other applications, as illustrated in Figure 3.

#### 5.1.1. Bioconversion of SCG into Prebiotic Manno-Oligosaccharides

SCG has potential for developing functional foods by producing prebiotic manno-oligosaccharides (MOS) and utilizing them as a substrate in the end products. These MOS have antioxidant, anti-inflammatory, and anti-cancer effects. The prebiotics produced from SCG were used to grow lactic acid bacteria under gastrointestinal stress for up to 2 h and showed an antibacterial effect by inhibiting the growth of *S. paratyphi* by up to 12.55%. As it resists gastrointestinal stress and promotes lactic acid bacteria growth, it can be used in dairy products, including yoghurt, which promotes the growth of probiotic bacteria. These effects support the growth and biofilm formation of five probiotic bacterial strains [35]. The extracted MOS can also be directly incorporated into the functional food products, including jellies, bakery goods, and functional beverages [76]. All these studies demonstrate the potential of SCG as a credible source to produce prebiotics for promoting gut and colonic health.

#### 5.1.2. SCG in Nutraceutical Formulations

SCG has been recognized as a promising source for the production of nutraceuticals due to its high levels of dietary fibres, polysaccharides, melanoidins, chlorogenic acid, and caffeoylquinic acid, demonstrating greater antioxidant, anti-inflammatory, and antimicrobial activity, and possessing significant health benefits [16]. Recent studies demonstrated that SCG demonstrates anti-adipogenic activity by inhibiting lipid accumulation through chlorogenic acid. In a comparative study of fermented and unfermented SCG, fermentation degraded chlorogenic acid, thereby decreasing anti-adipogenic activity [76]. Ali et al. (2018) formulated nutraceutical biscuits using dried SCG for obesity-related and diabetes issues by incorporating around 2–6% of SCG in wheat flour, which has dietary insoluble fibres, proteins, and lower glycemic sugars [77]. Additionally, the chemical compounds of SCG exhibit antimicrobial effects, antihypertensive effects, antimutagenic effects, antiallergenic effects and anticancerogenic effects, which can be used for nutraceutical formulation [5].

#### 5.1.3. Fermented Foods from SCG

SCG can be utilized as a good substrate to produce alcoholic beverages and organic acids through microbial fermentation. The addition of a monoculture of *Saccharomyces cerevisiae* and *Lachancea thermotolerans* (5% *v*/*v*) to SCG and incubating it at 20 °C for 3 days enables the production of alcoholic beverages. The incorporation of 0.25% (*w*/*v*) yeast extracts into those fermented hydrolysates enhanced the growth of cultures, flavour compounds, and increased organic acid production [78]. A similar study demonstrated that the addition of 0.25% (*w*/*v*) yeast extract to the non-saccharomyces wine yeasts, *Toeulaspora delbrueckii* and *Pichia kluyveri*, increased ethanol production and odour compounds, which has shown great potential in the development of beverages, but it reduced chlorogenic and caffeic acids [79]. When it comes to the sensorial perception of the beverage prepared from SCG hydrolysates, using fermentation and distillation provides a pleasant smell and coffee taste, making it acceptable according to human organoleptic properties. Through acidogenic fermentation, SCG can be converted into high-value short-chain organic acids. These fatty acids can be used as acidity regulators, flavouring agents, and natural preservatives in the bakery and beverage industry for industrial processing [32].

#### 5.1.4. Protein Recovery and Single-Cell Protein Production from SCG

SCG can be used as a promising sustainable alternative substrate to produce proteins; these proteins are high-value substances as they can be used in numerous industrial applications, including cosmetics, food, and pharmaceuticals. Conversely, protein extraction from SCG can be difficult owing to their complex matrix; successful purification and utilization can lead to innovative applications, including functional foods and create a circular economy. Recent studies demonstrated that with acid extraction followed by alkaline precipitation and alkaline extraction followed by acid precipitation, the recovery of proteins was 84.5 and 75.9%, respectively [80]. Alongside protein extraction, this SCG can also be used as a substrate for the development of single-cell proteins. Jomnonkhaow et al. (2024) produced yeast-based single-cell proteins by using hydrolyzed SCG as a carbon source [43]. Additionally, the extracted protein from SCG shows higher anti-hypertensive potential.

#### 5.1.5. SCG as a Functional Ingredient in Value-Added Foods

The development of functional foods by incorporating SCG into bakery foods, dairy products, and meat products has been studied in numerous ways. The addition of SCG adds bulk to the material and improves its functional properties because these are rich in bioactive compounds, which have higher antioxidant activities. The direct incorporation of dried SCG powder into cheese whey, after spray drying together, can be used as a stabilizing agent in high-fat mixtures. It can also be used as an emulsion agent, a foaming agent, and as a functional ingredient in baked goods and dairy products like yoghurts and ice creams [81]. The extracts of SCG with higher antioxidant activity can be used as an antioxidant agent for preserving both cooked and raw meat by applying them as a coating on their outer surfaces. The dried SCG powder can be directly incorporated into cookies, with an addition of around 7%, enhancing the TPC and shelf-life up to 471 days [82]. Addition of SCG to beef patties enriched dietary fibre and antioxidants, and a good point to consider is the higher sensorial acceptance for the SCG incorporated patties than the control [83]. All these studies represent the variability of SCG as a functional ingredient in food applications by acting as a good source of bioactive and antioxidant activity and supporting health.

#### 5.1.6. SCG as Fat Alternatives

SCG contains oil with a higher amount of antioxidants and phenolic compounds; using this as an alternative to fat will act as a healthier and vegan alternative to butter. It can be used in baked goods and gel formations. Cookies developed with 20% substitution of butter with SCG oil had a good texture with higher antioxidant activity [84]. SCG has potential for oleogel formation through the addition of SCG oil in whey protein in the ratio of 1:5, forming soft-textured gels, highlighting its potential as a fat replacer in food applications, including bakery and confectionery foods. A recent study demonstrated the potential of SCG as a lipid source for the synthesis of wax ester and the formation of oleogel by olive oil. This supports the application of functional fat mimetics and also shows the potential of the circular economy through sustainable utilization [85].

#### 5.1.7. Development of Packaging Films

SCG is rich in polysaccharides, making it a good source for the development of biodegradable packaging films or edible coatings. The main polysaccharides present in SCG are cellulose and galactomannans, which act as fillers in the film matrix and provide good barrier properties. The film developed from the SCG polysaccharide fraction by crosslinking with calcium ions shows good barrier properties against the water vapour transmission [86]. The film developed by solubilizing SCG in ZnCl_2_ and crosslinking with CaCl_2_ shows barrier properties against water vapour, as well as the ability to prevent UV and IR radiation, and also degrades in soil within 100 days [87]. SCG can also be used as an antioxidant agent in polylactic acid-based film development to enhance shelf-life, and it can also improve gas barrier properties [88]. Using electrospinning or solvent casting, SCG can be used for active zein film, which will perform a dual function as a packaging material and sustainable valorization of waste [86]. It is also used as a protein source for mycelium-based packaging material, highlighting the potential of SCG as a multi-functional source.

### 5.2. Non-Food Applications

**Figure 4:**
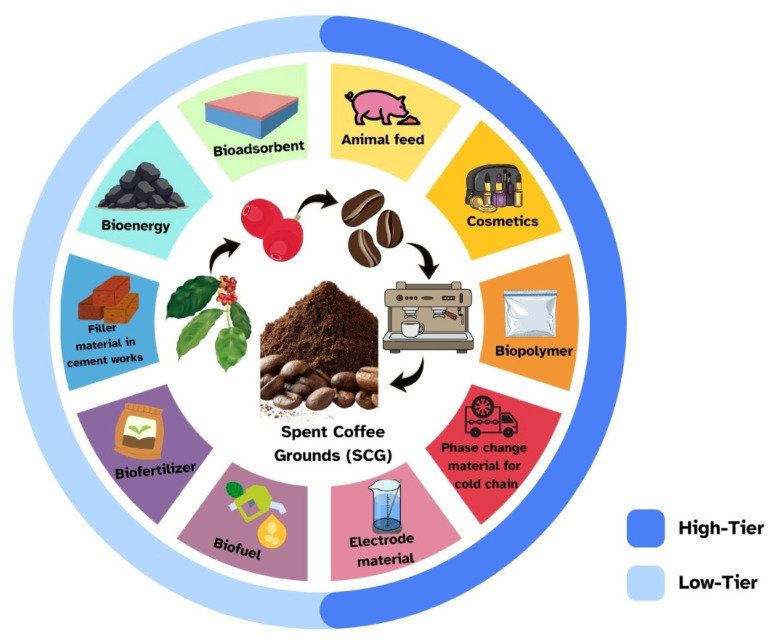
Overview of SCG potential in non-food valorization applications.

SCG has gained strong attention in numerous non-food applications due to their rich chemical composition, which aligns with the sustainable management of wastes and the potential of generating a circular economy (Figure 4). It can be used as animal feed, a source for biofuel, a soil amendment, in the cosmetic industry, as a biocatalyst, bioabsorbent, or bioplastic, as an electrode material, in the textile industry, as a phase change material, and in many other applications. SCG can be included at 3% and 2% of the total diet for sheep and cows, respectively. The pretreated and enzymatically hydrolyzed SCG has high nutritional value and improves the productivity of ruminants. A recent study reported that feeding 200 g/kg dry matter of SCG to dairy sheep resulted in the reduction of methane emission and improved the fatty acid profile without affecting the milk yield and the nutritional composition of milk [89]. It can be utilized as a replacement for palm kernel cake by up to 50% in the goat diet, significantly improving average body weight and even enhancing digestibility [90]. In the ruminant diet, it can be included as a protein source at 50–75%, and all these studies indicate the potential of SCG as a low-cost, sustainable alternative feed ingredient for animals. In the cosmetic industry, the usage of SCG oil has played a vital role because it contains linoleic acid, oleic acid, steric acids, and palmitic acid in major portions, all of which have significant functions like anti-inflammatory properties, preventing skin redness and hyperpigmentation, and protection against ultraviolet rays due to its high antioxidant activity [16].

It was reported that, compared to coffee oil, SCG oil has high antioxidant activity and does not have a negative effect on skin cell structure when used. SCG can be used to prepare the base of a cosmetic scrub by including other emulsifying agents and natural exfoliants to remove dead cells, highlighting SCG as a sustainable alternative to conventional cosmetic materials [91].

The most researched area in SCG valorization is the conversion of SCG into bioenergy through various techniques, including densification, pelletization, briquetting, torrefaction, transesterification, and anaerobic digestion, showing potential in the generation of a circular economy [54]. In a comparative study of the production of biofuel from the SCG through transesterification and pyrolysis, pyrolysis proved to be an effective method from an economic point of view, but in transesterification, the amount of CO_2_ emission is reduced [92]. After biofuel production, the residues can be used to generate activated carbon, which shows good absorption rates. Through pyrolysis, high-energy yields can be achieved. SCG has proven to be a good feedstock material for producing sustainable bioenergy. The soil fertility can be improved sustainably using SCG, due to its rich composition of carbohydrates, proteins, lipids and bioactive compounds. At very low concentrations, SCG will restrict the growth of plants due to the competition for soil nitrogen, so vermicomposting and pyrolysed SCG addition to soil will significantly improve the yields [18]. In the textile industry, for the development of biosorbents for dye removal, SCG can be used as a substrate for the production of fungal laccase, and the obtained mixture can be utilized as a biosorbent [8]. The carbon derived from SCG has significant potential in the development of a low-cost, sustainable alternative for the electrode material, which has good electrochemical properties and can be utilized according to its electrical conductivity and stability in capacitors, batteries, and electrochemical sensors [93].

For wastewater treatment, direct utilization of pyrolyzed SCG shows less effectiveness, but the addition of iron salt as a catalyst during the carbonization process improves the efficiency by providing absorbent spots. In the application of SCG as an adsorbent, it can be used as a flow reactor for treating wastewater from the textile industry by installing it as five serially connected reactors with nitric acid as a solvent [94]. These are also used as filler material in civil construction by incorporating them in vinyl ester using vacuum bagging [95]. An interesting application of SCG in the non-food sector is using it as a phase change material. The extracted oil can be used as a phase change material with a phase change temperature of 4.5 ± 0.72 °C, maintaining this temperature for up to 46 min, and can be a sustainable alternative in cold-chain storage applications [96]. All these approaches for valorizing SCG will help to reduce the environmental risk, increase the production of high-value products from waste, and generate a circular economy.

## 6. Other Considerations: Safety, Techno-Economic and Sustainability

The large-scale valorization of SCG requires an in-depth assessment of its safety, regulatory compliance, economic viability, and environmental sustainability, in addition to its technical viability. These concerns are of prime importance for ensuring the safe utilization of SCG in various applications without compromising economic and ecological balance.

### 6.1. Safety and Regulatory Considerations of SCG

SCG, despite its applicability as a functional food ingredient, should be thoroughly evaluated in terms of its safety, as it has potential contaminants. Conditional heavy metals like lead (Pb), cadmium (Cd) and arsenic (As) could build up in SCG depending on cultivation conditions and exposure to the environment; therefore, analyzing heavy metals is necessary on a regular basis. Moreover, process contaminants such as acrylamide, polycyclic aromatic hydrocarbons, furans, and other products of the Maillard reaction can form during coffee roasting, which may be retained in SCG, generally with a low level of acrylamide content below the threshold [13]. In addition, there is a potential risk of residual pesticides arising during cultivation. Biological risks are also a factor to consider, including the occurrence of mycotoxins due to poor handling of coffee beans during post-harvest. SCG usually has a high moisture content of 50–60% after brewing, which provides a favourable environment for moulds and bacteria that are prone to spoilage, due to improper handling during drying, transportation and storage [97]. There is also significant concern about the migration of induced contaminants when SCG is used in packaging film development. Therefore, it is crucial to conduct migration testing and follow food-contact regulations to guarantee that packaging materials do not release harmful substances exceeding established safety limits [98]. From a regulatory point of view, SCG are not recognized as Generally Recognized as Safe by the U.S. Food and Drug Administration, so their overall safety for use in food systems requires extensive safety validation [99]. For animal feed applications, SCG must comply with EU feed regulations, ensuring safety, traceability, and absence of contaminants. The EU also focuses on caffeine content and the safety of novel food additives [100].

### 6.2. Techno-Economic Analysis

Techno-economic analysis (TEA) is also very important in assessing the industrial feasibility of SCG valorization processes. SCG has a low-cost advantage in raw materials as compared to conventional feedstocks, but processing costs have a great impact on overall feasibility. Various methods of extraction, like Soxhlet, ultrasound-assisted extraction (UAE), and supercritical fluid extraction (SFE), vary in the cost–yield correlations. Capital expenditure (CAPEX) and operational expenditure (OPEX) are highly dependent on the configuration of the process, size and volume of the solvent used. Research on the production of activated carbon by SCG showed that OPEX ranges between 5.9 and 11.4 million USD per operation based on the process complexity. Chemical inputs and utilities playing a significant role in raising the cost of operation. In addition, the scalability of SCG-based biofuel production is economically constrained by the high drying costs associated with the presence of high moisture (i.e., 50–60%) [101]. Also, other logistics related to collection, transportation and storage influence feasibility. SCG valorization through integrated biorefinery enhances total economics. The techno-economic considerations of SCG valorization, including CAPEX and OPEX expenses, yield and process bottlenecks, are relatively tabulated in Table 5.

### 6.3. Life Cycle Assessment

It is necessary to conduct a life cycle assessment (LCA) to evaluate the environmental sustainability of SCG valorization pathways. In contrast to traditional disposal methods such as landfill and composting, SCG valorization may result in a significant decrease in greenhouse gas emissions and the level of fossil resource utilization by transforming waste into value-added products. As an illustration, the use of SCG in bio-based composites reduced the cumulative energy requirement and the carbon footprint by about 7–8% [109]. Nevertheless, LCA studies indicate significant environmental trade-offs, especially those related to energy-intensive drying, transportation logistics, and processing. Pelletization and conversion procedures have environmental effects, whereas transport distance has a strong impact on general sustainability [110]. These phases are regarded as key environmental hotspots. Conversely, disposal processes such as landfills result in methane release and a lack of biomass potential. All in all, the integrated valorization strategies benefit the environment; however, the focus on energy consumption and logistics is essential to ensure a sustainable approach.

## 7. Conclusions

SCG are a high-nutrient agro-industrial by-product with a lignocellulosic composition, with significant prospects for valorization in the food, nutraceutical, pharmaceutical, and bioenergy industries. It includes high concentrations of insoluble dietary fibre, lipids, proteins, minerals and phenolic bioactive substances, which offer functionalities and antioxidants that can be used in the development of value-added products. Their addition to food formulations such as bakery goods, snack foods, beverages and functional foods has shown advancement in nutritional enrichment, dietary fibre boosting and creating value along with food products. Besides direct use in food, the bioactive compounds of SCG have potential applications in nutraceutical formulations, and the biomass left after use can be reused for biofuel production, biodegradable materials, and agricultural amendments to enhance integrated waste valorization strategies. Though limitations regarding compositional variability, safety analysis and storage stability still exist, continuing development of pretreatment, stabilization and extraction technology is enhancing the possibility of SCG application. The use of pilot-scale processing, techno-economic evaluation, and circular bioeconomy plans also promotes the industrial implementation of SCG-based products. Commercialization will be faster in the future with processing standardization, regulatory compliance, and optimization of SCG functionality in complex food matrices. Overall, SCG valorization is a scientifically sound and environmentally friendly approach that aids in recovering resources and minimizing agro-industrial wastes, developing novel functional food components, and allowing non-food industrial purpose.

## 8. Emerging Trends and Future Directions

Future research on SCG will focus on the shift to integrated, intelligent bioprocessing systems. Innovative methods, such as precision fermentation with engineered microorganisms, are being investigated to optimize the selective conversion of sugars in SCG into high-value metabolites, improving process performance. Multi-product cascading biorefinery systems are also becoming popular, which allows simultaneous recovery of polyphenols, lipids, oligosaccharides and biofuels, in turn maximizing resource use and economic viability. AI-driven optimization tools for process extraction and design of the process are also beginning to appear, which enable the prediction of the optimal conditions and improvement of the yield. Moreover, the production of NADES systems capable of providing scalable food-grade purification technologies is also essential to allow the system to be used in industries. Interconnection of SCG valorization in coffee shop waste networks provides a decentralized and sustainable feedstock chain.

The future of SCG valorization is expected to involve advancements in process intensification and energy-efficient technologies to overcome challenges in SCG valorization, such as those posed by its moisture content. Continuous processing technologies are also expected to be used in SCG valorization. This is likely to improve its scalability and industrialization. Further, SCG valorization is expected to focus on the development of advanced biomaterials, carbon-based materials, and sustainable packaging. There is also expected to be an emphasis on standardization, storage stability, and optimization of SCG feedstocks. These are expected to contribute to the efficient and sustainable valorization of SCG.

As far as safety is concerned, comparative risk assessment is not over-researched. Even though SCG contains useful bioactives, the issues of residual contaminants, anti-nutritional aspects and chronic exposure will require that threshold levels of safe SCG content be established in food systems [111]. Existing toxicological information is scant, and extensive risk–benefit studies on chronic intake are nonexistent. There are still some serious gaps in the knowledge. Standardized protocols for SCG characterization do not exist, hence the varying results in composition and processing. There is a lack of toxicological information, especially on long-term dietary exposure. In addition, limited experiments have confirmed multi-step biorefinery models at the pilot or industrial scale, and conflicting results have been found regarding the bioactive stability of processing. There is very little information on consumer acceptance and sensory integration of complex food matrices. There are no regulatory pathways since there are no formal submissions. The solution to these gaps is to pursue interdisciplinary studies that combine process engineering, toxicology, regulatory science and food technology to facilitate safe and scaled SCG valorization.

## Figures and Tables

**Table 1:** Nutritional composition of SCG.

Component	Content	Key Constituents
Carbohydrates	45–56%	Polysaccharides, including cellulose (10–20%), hemicellulose (39–40%), and galactomannans rich in mannose (21–46%) and galactose (15–32%)
Dietary Fibre	Insoluble: ~35% Soluble: ~8%	Structural fibre components
Lignin	22.2–33.6%	Coniferyl, *p*-coumaryl, sinapyl alcohols
Lipids	18–25%	Linoleic acid (36–42%), Palmitic acid (~32%), Oleic acid (10–12%), Stearic acid (7–10%)
Proteins	10–17.5%	Amino acid-rich fraction
Phenolic compounds	16–173.3 mg GAE/g DW	Chlorogenic, caffeic, gallic, ferulic, protocatechuic acids; flavonoids (quercetin, catechin, kaempferol)
Minerals (mg/kg)	mg/kg	K (~11,700), P (1800), Mg (1900), Ca (1200), S (1600), Fe (52), Mn (29), Cu (19), Co (15), Zn (8)

Notes: Values are reported as ranges from the literature. GAE = gallic acid equivalents.

**Table 2:** Factors influencing the composition and recovery of target compounds from SCG.

Factor	Target Component Affected	Effect Observed	Possible Mechanism	Reference
Roasting degree	Chlorogenic acid	Decreases	Thermal degradation and Maillard reactions	[19]
Roasting degree	Melanoidins	Increases	Maillard reactions	
Brewing method	Caffeine, phenolics	Higher retention in hot brew than in cold brew	Temperature-dependent	[20]
Coffee variety (Arabica/Robusta)	Caffeine, phenolics	Robusta contains higher caffeine	Genetic variation in bean composition	
Geographical origin	Antioxidants, phenolics	Significant variation	Differences in climate, altitude, and soil	
Grinding size	Phenolic extraction	Finer particles increase extraction	Increased surface area	[21]
Extraction pressure/temperature	Lipids and antioxidants	Higher recovery	Enhanced mass transfer	

Note: The observed effects may vary depending on coffee processing conditions, brewing parameters, and extraction techniques.

**Table 3:** Pretreatment strategies for SCG and their impact on valorization efficiency.

Method of Pretreatment	Purpose of Pretreatment	Pretreatment Conditions	Effect of Pretreatment on SCG	Yield	References
Oven drying and defatting	To reduce the moisture content and the lipid content	45 °C for 24 h, Soxhlet extraction at 70 °C for 6 h	Improved solvent absorption and enhanced the yields of phenolic compounds	34.43 mg GAE/g DW SCG phenolic recovery	[28]
Supercritical CO_2_ deoiling	To remove the oil content from SCG	55 °C, 30 MPa, CO_2_ flow rate: 27 g/min, extraction time:2 h	Improved the recovery of flavonoids and antioxidant activity	TPC 35.58 ± 0.68 mg GAE/g DW; antioxidant activity: 64.9 mg TE/g	[29]
Ultrasound treatment	To break down the complex lignocellulosic structure and improve the mass transfer of the solvent	Liquid-to-solid ratio: 26.7 mL/g: 40 °C, 30 min, 400 W, 60 Hz	Increased the yields of reducing sugars and phenolic compounds with higher antioxidant activity	TPC 43.45 ± 0.28 mg GAE/g DW, TFC 64.28 ± 0.75 mg QE/g DW with 70.3 mg TE/g antioxidant activity	
Subcritical water extraction treatment	To enhance downstream hydrothermal carbonisation efficiency by extracting bioactives	Liquid-to-solid ratio: 30 mL/g, 180 °C for 30 min	Reduced the nitrogen content in hydrochar, improved the accessibility of proteins and quality of solid biofuel	Protein yields increased to 53%, and reduced nitrogen by 0.23%	[30]
Alkaline pretreatment	To remove the lignin content to enhance the enzymatic hydrolysis for prebiotic production	NaOH loading: 0.25 g/g SCG; liquid-to-solid ratio: 20 mL/g; 70 °C for 4 h	Increased the availability of mannan and removed the phenolics and lipids	MOS: 1.20 ± 0.04 mg/mL	[31]
Hydrothermal pretreatment	To enhance the acidogenic fermentation for monosaccharides extraction	121 °C for 1 h	Lignocellulosic structure ruptured, increasing the monosaccharide yields at mild conditions	Monosaccharides: 0.05 g/L Oxygen demand: 2.95 g COD/L TPC: 16.8 mg GAE/g DW	[32]
Drying and solvent pretreatment	To improve the structure to act as a catalyst for biodiesel production from the waste cooking oil	Dried at 100 °C overnight, and acetone is used as the solvent, washed for 45 min	Increased the particle surface area and increased the reactivity of SCG	SCG exhibited good catalytic properties and yielded biodiesel up to 92.31%	[33]
Mild alkaline pretreatment	Delignification	0.625 M NaOH, at 50 °C for 60 min	The complex lignin structure was broken down, enhancing the accessibility for the biorefinery approach	The yields of lignin & oligosaccharides were 6.25 kg and 6.36 kg per 100 kg of SCG	[34]

Notes: TPC = total phenolic content; TFC = total flavonoid content; GAE = gallic acid equivalents; COD = chemical oxygen demand; MOS = manno-oligosaccharides. Values are reported as obtained under specific experimental conditions from respective studies.

**Table 4:** Overview of food and non-food valorization routes of SCG.

SCG Characteristics	Valorization Technique	Treatment Conditions	Application	Key Output	References
SCG extracts	Solvent extraction	Sample-to-solvent ratio: 1:40; 50 °C; 1 h; 200 rpm	Natural preservative for shelf-life extension of minced beef	0.5% SCG prevented lipid oxidation by 52.47% by the 14th day in refrigerated conditions and also improved sensory properties	[68]
Freeze-dried SCG	Micro-solid phase extraction	pH adjusted to 2 and µ-SPEed using the PS/DVB-RP sorbent, MeOH activation	Recovery of bioactive compounds with high antioxidant activity	The yields of bioactive compounds were 53.7 ± 3.1 mg GAE/100 g DW with antioxidant activity of 78.1 ± 7.3 mg TE/100 g DW	[24]
Dried SCG powder	Powder substitutions in bakery products	SCG incorporated as rice flour substitute for gluten-free cake formulation at 0%, 25%, 50%, 75%, and 100%	As a functional ingredient in the development of gluten-free cake	Phenolic content 2.70 mg GAE/g DW with antioxidant activity of 4.17 mg TE/g improved volume at 25% and enhanced the sensory acceptability at 25–50% SCG incorporation	[69]
Dried SCG	Enzymatic hydrolysis	Endo-β-mannanase treatment at 65 °C for 72 h, and a cocktail was produced with 5.5 pH	Production of functional foods with prebiotic saccharides	SCG to 52% polysaccharides conversion, reducing sugars 17.4 mg/50 mg of SCG, Mos’s yield of 62.3 mg/500 mg of SCG	[35]
Dried SCG	Sulfuric acid treatment and centrifugation of hydrolysates	2% H_2_SO_4,_ 20 min, and centrifuged at 1157× *g* for 10 min	Recovery of oligosaccharides for functional and prebiotic applications	Extracted OS yields up to 22.4 g/100 g of SCG with hexose and pentose chains with up to eight sugar units	[17]
SCG with sand-like granular structure	Biopolymer composite development	SCG mixed with NaOH-casein binder, moulded and cured at 20–50 °C, for 7–90 days	Sustainable bio-composite material for construction and circular economy applications	Biopolymer with a strength of 7.7 MPa and it improved the structural stability	[70]
Dried SCG	Microwave-assisted pyrolysis (MAP) followed by densification	MAP at 300–400 °C for 30–90 min and densification	Sustainable solid biofuel as alternative for coal alternative	Carbon content increased to 39.97% with calorific value 27.10 MJ/kg with 16.54 min combustion duration	[71]
Defatted SCG	Polymerization of SCG lipids, acidification of defatted SCG and PLE for caffeine recovery	Polymer curing with phthalic acid at 180–220 °C for 3–24 h, citric acid used for treating defatted SCG	Sustainable bio-based polymer and filler material	Polymer with 50 g/m^2^ water vapour transmission and thickness of 0.6–0.7 mm	[72]
Dried SCG	Hydrothermal carbonization	Acidification with H_2_SO_4_ and hydrothermal treatment at 200 °C for 12 h	Biosorbent for wastewater treatment	H_2_O_2_ yield up to 610 µmol/L and efficiently purified wastewater	[73]
SCG extract	Green synthesis of ZnO nanoparticles using SCG extract	1:10 S/L ratio extraction at 80 °C for 60 min and treated with zinc acetate, calcinated at 600 °C	Biocompatible nanomaterial production	Developed stable hexagonal ZnO nanoparticle with 5–20 nm	[74]
SCG	Hydrothermal treatment	Hydrothermal treatment at 200 °C for 12 h, dialysis, drying at 80 °C and compression moulding	Solar-driven hydrothermal-treated SCG evaporator	High evaporation rate of 2.45 kg/m^2^, freshwater yield of 7.14 kg/m^2^/h	[73]
Dried SCG	Pyrolysis	Pyrolysed at 500 °C, treated with magnesium oxide and calcination	Biosorbent for fluorine removal from groundwater	Fluoride was removed with the 86% efficiency and an adsorption capacity of 141.98 mg/g	[75]
Fresh SCG	Hydrodynamic cavitation	Hydrodynamic cavitation with 3/10 mm orifice plate, 3–7 bar pressure for 5–45 min	Improving SCG biodegradability for anaerobic digestion and wastewater treatment	Organic carbon ratio improved up to 71%, enhanced degradability and observed caffeine release at 5–7 bar	[37]

Notes: GAE = gallic acid equivalents; TE = Trolox equivalents; OS = oligosaccharides; PLE = pressurized liquid extraction; MAP = microwave-assisted pyrolysis. Values are reported as obtained under specific experimental conditions from respective studies.

**Table 5:** Techno-economic considerations of SCG valorization.

Extraction Technique	CAPEX	OPEX	Yield vs. Cost	Limitation for Commercial Adoption	Reference
Soxhlet extraction	Low	High	Moderate yield but high cost per unit volume	Solvent recovery, longer durations	[102]
Ultrasound-assisted extraction	Medium	Low-Medium	High yields but reduced cost per unit	Scale-up limitation	[103]
Supercritical Co_2_ extraction	High	Medium	High yield with high purity and high cost	High equipment cost, pressure requirements	[104]
Pressurized liquid extraction	High	Medium-High	High extraction efficiency but high cost	Thermal degradation, equipment cost	[105]
NADES	Medium	Medium	Good recovery with moderate cost	Solvent recovery	[106]
Fermentation	Medium	High	Variable yields due to inconsistent cost efficiency depending on substrate conversion rates	Process control, contamination issues	[107]
Integrated approach	Very high	Medium	High yields but reduced cost per product due to multi-stream valorization	Complex design, scalability	[108]

Notes: CAPEX = capital expenditure; OPEX = operating expenditure; NADES = natural deep eutectic solvents. Cost and yield assessments are qualitative and based on literature-reported trends.

## Data Availability

No new data were created or analyzed in this study. Data sharing is not applicable to this article.

## References

[1] Sidło W., Latosińska J. (2025). Reuse of Spent Coffee Grounds: Alternative Applications, Challenges, and Prospects—A Review. Appl. Sci..

[2] USDA Agricultural Projections to 2025|Economic Research Service. https://www.ers.usda.gov/publications/37818.

[3] International Coffee Organization. https://ico.org/.

[4] Zhao N., Liu Z., Yu T., Yan F. (2024). Spent coffee grounds: Present and future of environmentally friendly applications on industries-A review. Trends Food Sci. Technol..

[5] Bevilacqua E., Cruzat V., Singh I., Rose’Meyer R.B., Panchal S.K., Brown L. (2023). The Potential of Spent Coffee Grounds in Functional Food Development. Nutrients.

[6] Coffee Consumption by Country 2026. https://worldpopulationreview.com/country-rankings/coffee-consumption-by-country.

[7] Bomfim A.S.C.d., Oliveira D.M.d., Voorwald H.J.C., Benini K.C.C.d.C., Dumont M.-J., Rodrigue D. (2022). Valorization of Spent Coffee Grounds as Precursors for Biopolymers and Composite Production. Polymers.

[8] França E.d.S., de Souza A.F., Rodríguez D.M., de Paula N.Z., Neves A.G.D., Cardoso K.B.B., Campos-Takaki G.M.d., de Lima M.A.B., Porto A.L.F. (2025). Valorization of Spent Coffee Grounds as a Substrate for Fungal Laccase Production and Biosorbents for Textile Dye Decolorization. Fermentation.

[9] Andrade C., Perestrelo R., Câmara J.S. (2022). Valorization of Spent Coffee Grounds as a Natural Source of Bioactive Compounds for Several Industrial Applications—A Volatilomic Approach. Foods.

[10] Sharma A., Ray A., Singhal R.S. (2021). A biorefinery approach towards valorization of spent coffee ground: Extraction of the oil by supercritical carbon dioxide and utilizing the defatted spent in formulating functional cookies. Futur. Foods.

[11] Piasek A.M., Bardadyn P., Trojan Z., Jelonek K., Wysocki Ł., Kobiela T., Sobiepanek A. (2025). Research on Spent Coffee Grounds: From Oil Extraction to its Potential Application in Cosmetics. Waste Biomass Valorization.

[12] de Bomfim A.S.C., de Oliveira D.M., Walling E., Babin A., Hersant G., Vaneeckhaute C., Dumont M.-J., Rodrigue D. (2022). Spent Coffee Grounds Characterization and Reuse in Composting and Soil Amendment. Waste.

[13] da Costa D.S., Albuquerque T.G., Costa H.S., Bragotto A.P.A. (2023). Thermal Contaminants in Coffee Induced by Roasting: A Review. Int. J. Environ. Res. Public Health.

[14] Ahmed H., Abolore R.S., Jaiswal S., Jaiswal A.K. (2024). Toward Circular Economy: Potentials of Spent Coffee Grounds in Bioproducts and Chemical Production. Biomass.

[15] Battista F., Barampouti E.M., Mai S., Bolzonella D., Malamis D., Moustakas K., Loizidou M. (2020). Added-value molecules recovery and biofuels production from spent coffee grounds. Renew. Sustain. Energy Rev..

[16] Quijote K.L., Go A.W., Agapay R.C., Ju Y.H., Angkawijaya A.E., Santoso S.P. (2021). Lipid-dense and pre-functionalized post-hydrolysis spent coffee grounds as raw material for the production of fatty acid methyl ester. Energy Convers. Manag..

[17] Fogarin H.M., Murillo-Franco S.L., Santos M.C.M., Silva D.D.V., Dussán K.J. (2025). Acid hydrolysis pretreatment for extraction of oligosaccharides derived from spent coffee grounds: Valorization of a promising biomass. Environ. Sci. Pollut. Res..

[18] Pérez-Burillo S., Cervera-Mata A., Fernández-Arteaga A., Pastoriza S., Rufián-Henares J.Á., Delgado G. (2022). Why Should We Be Concerned with the Use of Spent Coffee Grounds as an Organic Amendment of Soils? A Narrative Review. Agronomy.

[19] Maiyah N., Kerdpiboon S., Kerr W.L., Klaypradit W., Smithisukul C., Supapvanich S. (2026). Impact of roasting levels and brewing cycles on bioactive compounds in spent coffee grounds. Food Chem. X.

[20] Głowacka R., Derewiaka D., Gorska A., Wirkowska-Wojdyła M., Wołosiak R., Majewska E. (2019). The influence of brewing method on bioactive compounds residues in spent coffee grounds of different roasting degree and geographical origin. Int. J. Food Sci. Technol..

[21] Bułkowska K., Zielińska M. (2025). Enhanced Anaerobic Digestion of Spent Coffee Grounds: A Review of Pretreatment Strategies for Sustainable Valorization. Energies.

[22] Maiyah N., Kerdpiboon S., Supapvanich S., Kerr W.L., Sriprom P., Chotigavin N., Klaypradit W., Puttongsiri T. (2025). Recovering bioactive compounds and antioxidant capacity of medium roasted spent coffee grounds through varied hydrothermal brewing cycles. J. Agric. Food Res..

[23] Yust B.G., Rao N.Z., Schwarzmann E.T., Peoples M.H. (2022). Quantification of Spent Coffee Ground Extracts by Roast and Brew Method, and Their Utility in a Green Synthesis of Gold and Silver Nanoparticles. Molecules.

[24] Andrade C., Perestrelo R., Câmara J.S. (2022). Bioactive Compounds and Antioxidant Activity from Spent Coffee Grounds as a Powerful Approach for Its Valorization. Molecules.

[25] Monente C., Ludwig I.A., Stalmach A., De Peña M.P., Cid C., Crozier A. (2015). In vitro studies on the stability in the proximal gastrointestinal tract and bioaccessibility in Caco-2 cells of chlorogenic acids from spent coffee grounds. Int. J. Food Sci. Nutr..

[26] Campos-Vega R., Vázquez-Sánchez K., López-Barrera D., Loarca-Piña G., Mendoza-Díaz S., Oomah B.D. (2015). Simulated gastrointestinal digestion and in vitro colonic fermentation of spent coffee (*Coffea arabica* L.): Bioaccessibility and intestinal permeability. Food Res. Int..

[27] Vázquez-Sánchez K., Martinez-Saez N., Rebollo-Hernanz M., del Castillo M.D., Gaytán-Martínez M., Campos-Vega R. (2018). In vitro health promoting properties of antioxidant dietary fiber extracted from spent coffee (*Coffee arabica* L.) grounds. Food Chem..

[28] Solomakou N., Loukri A., Tsafrakidou P., Michaelidou A.M., Mourtzinos I., Goula A.M. (2022). Recovery of phenolic compounds from spent coffee grounds through optimized extraction processes. Sustain. Chem. Pharm..

[29] Park J.S., Nkurunziza D., Roy V.C., Ho T.C., Kim S.Y., Lee S.C., Chun B.S. (2022). Pretreatment processes assisted subcritical water hydrolysis for valorisation of spent coffee grounds. Int. J. Food Sci. Technol..

[30] Massaya J., Chan K.H., Mills-Lamptey B., Chuck C.J. (2023). Developing a biorefinery from spent coffee grounds using subcritical water and hydrothermal carbonisation. Biomass Convers. Biorefin..

[31] Magengelele M., Malgas S., Pletschke B.I. (2023). Bioconversion of spent coffee grounds to prebiotic mannooligosaccharides—An example of biocatalysis in biorefinery. RSC Adv..

[32] Pereira J., de Melo M.M.R., Silva C.M., Lemos P.C., Serafim L.S. (2022). Impact of a Pretreatment Step on the Acidogenic Fermentation of Spent Coffee Grounds. Bioengineering.

[33] Silvaraja J., Yahya N.Y., Zainol M.M., Lee Y.S. (2025). Preliminary investigations of sustainable magnetic catalyst-based biochar derived spent coffee ground for biodiesel production from waste cooking oil. Clean. Chem. Eng..

[34] Ribeiro G.M., Martins P.L., Oliveira A.C., Carvalheiro F., Fragoso R., Duarte L.C. (2023). The Role of Mild Alkaline Pretreatment in the Biorefinery Upgrade of Spent Coffee Grounds. Energies.

[35] Shaikh-Ibrahim A., Curci N., De Lise F., Sacco O., Di Fenza M., Castaldi S., Isticato R., Oliveira A., Aniceto J.P.S., Silva C.M. (2025). Carbohydrate conversion in spent coffee grounds: Pretreatment strategies and novel enzymatic cocktail to produce value-added saccharides and prebiotic mannooligosaccharides. Biotechnol. Biofuels Bioprod..

[36] Arauzo P.J., Lucian M., Du L., Olszewski M.P., Fiori L., Kruse A. (2020). Improving the recovery of phenolic compounds from spent coffee grounds by using hydrothermal delignification coupled with ultrasound assisted extraction. Biomass Bioenergy.

[37] Szaja A., Montusiewicz A., Pasieczna-Patkowska S., Grządka E., Montusiewicz J., Lebiocka M. (2024). Pre-Treatment of Spent Coffee Grounds Using Hydrodynamic Cavitation. Energies.

[38] Kyriakoudi A., Loukri A., Christaki S., Oliinychenko Y., Stratakos A.C., Mourtzinos I. (2024). Impact of Cold Atmospheric Plasma Pretreatment on the Recovery of Phenolic Antioxidants from Spent Coffee Grounds. Food Anal. Methods.

[39] Leal Vieira Cubas A., Medeiros Machado M., Tayane Bianchet R., Alexandra da Costa Hermann K., Alexsander Bork J., Angelo Debacher N., Flores Lins E., Maraschin M., Sousa Coelho D., Helena Siegel Moecke E. (2020). Oil extraction from spent coffee grounds assisted by non-thermal plasma. Sep. Purif. Technol..

[40] Ballesteros L.F., Cerqueira M.A., Teixeira J.A., Mussatto S.I. (2015). Characterization of polysaccharides extracted from spent coffee grounds by alkali pretreatment. Carbohydr. Polym..

[41] Cho E.J., Lee W.H., Lee Y.G., Van Ta Q., Kim H.Y., Bae H.J. (2025). Optimizing the treatment and valorization of spent coffee grounds (SCGs) through the integration of sequential pretreatment steps, enzymatic hydrolysis, and selective fermentation. Food Chem. X.

[42] Kim S., Kim J.C., Kim Y.Y., Yang J.E., Lee H.M., Hwang I.M., Park H.W., Kim H.M. (2024). Utilization of coffee waste as a sustainable feedstock for high-yield lactic acid production through microbial fermentation. Sci. Total Environ..

[43] Jomnonkhaow U., Plangklang P., Reungsang A., Peng C.Y., Chu C.Y. (2024). Valorization of spent coffee grounds through integrated bioprocess of fermentable sugars, volatile fatty acids, yeast-based single-cell protein and biofuels production. Bioresour. Technol..

[44] Arslan-Tontul S., Yang S., Casertano M., Pellegrini N., Fogliano V. (2026). Upcycling of spent coffee grounds solid-state fermentation by Aspergillus strains: In vitro assessment of prebiotic activity and gut health benefits. Food Chem..

[45] Samtiya M., Badgujar P.C., Chandratre G.A., Aluko R.E., Kumar A., Bhushan B., Dhewa T. (2024). Effect of selective fermentation on nutritional parameters and techno-functional characteristics of fermented millet-based probiotic dairy product. Food Chem. X.

[46] Jin L.S., Salimi M.N., Kamal S.Z. (2020). Optimization of Pretreatment and Enzymatic Hydrolysis of Spent Coffee Ground for the Production of Fermentable Sugar. IOP Conf. Ser. Mater. Sci. Eng..

[47] Bhaturiwala R.A., Modi H.A. (2020). Extraction of oligosaccharides and phenolic compounds by roasting pretreatment and enzymatic hydrolysis from spent coffee ground. J. Appl. Biol. Biotechnol..

[48] Kanaan A.F., dos Santos K.C., Menezes M.A.H., Hamerski F., Voll F.A.P., Corazza M.L. (2025). Sequential extraction of industrial spent coffee grounds using pressurized fluids as solvents. J. Supercrit. Fluids.

[49] Coelho J.P., Filipe R.M., Paula Robalo M., Boyadzhieva S., Cholakov G.S., Stateva R.P. (2020). Supercritical CO2 extraction of spent coffee grounds. Influence of co-solvents and characterization of the extracts. J. Supercrit. Fluids.

[50] Romano R., De Luca L., Basile G., Nitride C., Pizzolongo F., Masi P. (2023). The Use of Carbon Dioxide as a Green Approach to Recover Bioactive Compounds from Spent Coffee Grounds. Foods.

[51] Toda T.A., Visioli P.D.C.F., de Oliveira A.L., da Costa Rodrigues C.E. (2021). Conventional and pressurized ethanolic extraction of oil from spent coffee grounds: Kinetics study and evaluation of lipid and defatted solid fractions. J. Supercrit. Fluids.

[52] Christoforidis A., Mantiniotou M., Athanasiadis V., Lalas S.I. (2025). Caffeine and Polyphenolic Compound Recovery Optimization from Spent Coffee Grounds Utilizing Pressurized Liquid Extraction. Beverages.

[53] Yusoff I.M., Mat Taher Z., Rahmat Z., Chua L.S. (2022). A review of ultrasound-assisted extraction for plant bioactive compounds: Phenolics, flavonoids, thymols, saponins and proteins. Food Res. Int..

[54] Dari D.N., da Silva L.F., Júnior A.M.B.L., Freitas I.S., da Silva Aires F.I., dos Santos J.C.S. (2025). Spent coffee grounds: Insights and future prospects for bioenergy and circular economy applications. Green Technol. Sustain..

[55] Osorio Arias J.C., Quintero M., Velázquez S., Escobar J. (2023). A novel process line for the valorization of industrial spent coffee grounds through coffee roasting technology and ultrasonic-assisted extraction. J. Chem. Technol. Biotechnol..

[56] Ramadan M., Fadel A., Bahi A., Alajlani S., Al Neyadi M.S.S., Alshamsi E.S.H., Naser S., Shahbaz H.M., Samuel R., Ranneh Y. (2025). Optimizing Chlorogenic Acid Extraction From Spent Coffee Grounds: A Comparative Review of Conventional and Non-Conventional Techniques. Food Sci. Nutr..

[57] Fernandes F., Delerue-Matos C., Grosso C. (2025). Valorization of Spent Coffee Grounds: Comparing Phenolic Content and Antioxidant Activity in Solid-Liquid vs. Subcritical Water Extraction Methods. Biol. Life Sci. Forum.

[58] Karprakhon V., Sirisangsawang R., Kaewkroek K., Rojviroon T., Phetyim N., Sukpancharoen S. (2025). Optimization of combined subcritical water and CO2 extraction for enhanced phenolics and antioxidant activity from coffee byproducts. Sci. Rep..

[59] Lee J.W., Lee S.Y., Lee M.Y., Hong G.P. (2025). Subcritical water mediated conversion of spent coffee grounds into physiologically active biomaterials for utilization in food industry. LWT.

[60] Costa C., Marques M., Martins A.M., Gonçalves L., Pinto P., Ribeiro H.M., Marto J., Paiva A. (2025). Upcycling Spent Coffee Grounds into Bioactive Extracts Using New Natural Deep Eutectic Systems for Sustainable Topical Formulations. ACS Sustain. Chem. Eng..

[61] Tzani A., Lymperopoulou T., Pitterou I., Karetta I., Belfquih F., Detsi A. (2023). Development and optimization of green extraction process of spent coffee grounds using natural deep eutectic solvents. Sustain. Chem. Pharm..

[62] da Silva C.N., Ferreira C.R., Ribeiro B.D., Buarque F.S. (2026). Recovery of Phenolic Compounds and Proteins from Spent Coffee Grounds Using Eutectic Solvents. Separations.

[63] Ballesteros-Gómez A., Serrano-Crespín A., Rubio S. (2023). Supramolecular-solvent based extraction of hydroxytyrosol from brines of the processing of table olives. Sep. Purif. Technol..

[64] Torres-Valenzuela L.S., Plaza-Dorado J.L., Osorio-Arias J.C. (2025). Valorization of domestic spent coffee grounds: Modeling and optimizing the extraction of bioactive compounds with supramolecular solvents. Int. J. Food Prop..

[65] Yusufoğlu B., Kezer G., Wang Y., Ziora Z.M., Esatbeyoglu T. (2024). Bio-recycling of spent coffee grounds: Recent advances and potential applications. Curr. Opin. Food Sci..

[66] Kovalcik A., Obruca S., Marova I. (2018). Valorization of spent coffee grounds: A review. Food Bioprod. Process..

[67] Araujo M.N., dos Santos K.C., do Carmo Diniz N., de Carvalho J.C., Corazza M.L. (2022). A biorefinery approach for spent coffee grounds valorization using pressurized fluid extraction to produce oil and bioproducts: A systematic review. Bioresour. Technol. Rep..

[68] Ayadi D.Z., Chaari M., Elhadef K., Abid C., Ennouri M., Varzakas T., Smaoui S. (2026). Spent coffee grounds extract as an effective approach for prolonging the shelf life of minced beef: Chemometric modeling of quality attributes. Qual. Assur. Saf. Crops Foods.

[69] Tiryaki G., Nakilcioğlu E. (2026). Effect of spent coffee ground substitution on physical, chemical, and sensory attributes of gluten-free cake. Gıda.

[70] Mansour Khodja A., Pliya P., Rehab Bekkouche S., Eslami J. (2026). Valorization of spent coffee grounds and casein for a new bio-composite material. Dev. Built Environ..

[71] Sophiana I.C., Liem C.A., Raga A.F., Safira F.Y., Baharin N.S.K., Steven S., Hara H., Ida T., Riyadi F.A. (2026). Spent coffee grounds valorization via microwave-assisted pyrolysis and densification to produce bio-coke for solid biofuel applications. Biomass Bioenergy.

[72] Eiseman J., Williamson K., Banker T., Dey S., Zhao X., Chen D., Campanella O., Vodovotz Y., Hatzakis E. (2026). Multivalorization of spent coffee grounds towards a waste free solution. Biomass Bioenergy.

[73] Wang X., Yuan S., Feng B., Qiu X., Yu C., Lu W., Xu X., Hu Y., Shi Y. (2025). Spent coffee ground-derived hydrochar: An ecologically compatible material for solar-driven H2O2 production and wastewater purification. J. Colloid Interface Sci..

[74] Winiarska K., Klimek-Ochab M., Wilk Ł.J., Winiarski J. (2025). Green synthesis of ZnO nanoparticles using spent coffee extract: Comprehensive characterization and insights. Mater. Chem. Phys..

[75] Yu Y.L., Chen C.Y., Dhanasinghe C., Verpoort F., Surampalli R.Y., Chen S.C., Kao C.M. (2024). Development of modified MgO/biochar composite for chemical adsorption enhancement to cleanup fluoride-contaminated groundwater. J. Environ. Manag..

[76] Im A.E., Choi J., Park H.S., Nam S.H. (2025). Extraction of mannooligosaccharides from spent coffee grounds and its application for functional jelly with improved physical properties and immunomodulatory effect. J. Food Sci. Technol..

[77] Ali H.S., Mansour A.F., Kamil M.M., Hussein A.M.S. (2018). Formulation of nutraceutical biscuits based on dried spent coffee grounds. Int. J. Pharmacol..

[78] Liu Y., Yuan W., Lu Y., Liu S.Q. (2021). Biotransformation of spent coffee grounds by fermentation with monocultures of Saccharomyces cerevisiae and Lachancea thermotolerans aided by yeast extracts. LWT.

[79] López-Linares J.C., García-Cubero M.T., Coca M., Lucas S. (2021). A biorefinery approach for the valorization of spent coffee grounds to produce antioxidant compounds and biobutanol. Biomass Bioenergy.

[80] Kim J., Park Y., Shin J., Kim S., Kim H.L., Bae S. (2025). Sustainable protein extraction from spent coffee grounds using response surface methodology. Biomass Convers. Biorefin..

[81] Osorio-Arias J., Delgado-Arias S., Duarte-Correa Y., Largo-Ávila E., Montaño D., Simpson R., Vega-Castro O. (2020). New powder material obtained from spent coffee ground and whey protein; Thermal and morphological analysis. Mater. Chem. Phys..

[82] Papageorgiou C., Dermesonlouoglou E., Tsimogiannis D., Taoukis P. (2024). Enrichment of Bakery Products with Antioxidant and Dietary Fiber Ingredients Obtained from Spent Coffee Ground. Appl. Sci..

[83] Dilek N.M. (2025). Utilization of Spent Coffee Grounds as an Antioxidant Dietary Fiber in Beef Patties: Oxidative Stability, Texture Properties, and Molecular Docking. Food Sci. Nutr..

[84] Meerasri J., Sothornvit R. (2022). Novel development of coffee oil extracted from spent coffee grounds as a butter substitute in bakery products. J. Food Process. Preserv..

[85] Papadaki A., Mandala I., Kopsahelis N. (2025). Development and Characterization of Emulsion-Templated Oleogels from Whey Protein and Spent Coffee Grounds Oil. Foods.

[86] Mottola S., Drago E., Montella F., Firpo G., Campardelli R., De Marco I. (2026). From spent coffee residues to sustainable packaging: Zein-based active films produced via supercritical impregnation. J. Supercrit. Fluids.

[87] Zhang S., Zhang X., Wan X., Zhang H., Tian J. (2023). Fabrication of biodegradable films with UV-blocking and high-strength properties from spent coffee grounds. Carbohydr. Polym..

[88] Pettinato M., Bolla M., Campardelli R., Firpo G., Perego P. (2023). Potential Use of PLA-Based Films Loaded with Antioxidant Agents from Spent Coffee Grounds for Preservation of Refrigerated Foods. Foods.

[89] Medjadbi M., Goiri I., Atxaerandio R., Ruiz R., Charef S.E., Benhissi H., Ibarruri J., Iñarra B., Gutiérrez M., San Martin D. (2025). Effect of feeding increasing levels of spent coffee grounds on milk yield and composition, methane emissions, milk fatty acid profile and curd sensory characteristics in Latxa dairy sheep. J. Anim. Sci..

[90] Chukaew K., Srisuwan C., Inaoy P., Prasanpanich S., Rangubhet K.T., Kongmun P. (2025). The Effects of Using Spent Coffee Grounds as a Protein Source in the Concentrated Diet of Goats. Anim. Sci. J..

[91] Szaferski W., Janczarek M. (2025). Preparation of Cosmetic Scrub Bases from Coffee Waste and Eco-Friendly Emulsifiers. Cosmetics.

[92] Gu J., Lee A., Choe C., Lim H. (2023). Comparative study of biofuel production based on spent coffee grounds transesterification and pyrolysis: Process simulation, techno-economic, and life cycle assessment. J. Clean. Prod..

[93] Pagett M., Teng K.S., Sullivan G., Zhang W. (2023). Reusing Waste Coffee Grounds as Electrode Materials: Recent Advances and Future Opportunities. Glob. Chall..

[94] Naghdbishi E., Khorasani A.C. (2025). A zero-waste approach to spent coffee grounds valorization: Cellulose production in series recycle flow reactors with its effluent upcycling into adsorbents for textile wastewater treatment. Int. J. Biol. Macromol..

[95] Mohan A.M.A., Vignesh V., Nagaprasad N., Krishnaraj R. (2025). Mechanical and thermal behaviour of waste spent coffee ground filler reinforced vinyl-ester composites for civil construction applications. Sci. Rep..

[96] Jin Ong P., Leow Y., Yun Debbie Soo X., Hui Chua M., Ni X., Suwardi A., Kiang Ivan Tan C., Zheng R., Wei F., Xu J. (2023). Valorization of Spent coffee Grounds: A sustainable resource for Bio-based phase change materials for thermal energy storage. Waste Manag..

[97] Choe U. (2025). Valorization of spent coffee grounds and their applications in food science. Curr. Res. Food Sci..

[98] European Commission Regulation-1935/2004-EN-EUR-Lex. https://eur-lex.europa.eu/eli/reg/2004/1935/oj/eng.

[99] FDA Generally Recognized as Safe (GRAS)|FDA. https://www.fda.gov/food/food-ingredients-packaging/generally-recognized-safe-gras.

[100] Animal Feed—Food Safety—European Commission. https://food.ec.europa.eu/food-safety/animal-feed_en.

[101] Mukherjee A., Okolie J.A., Niu C., Dalai A.K. (2022). Techno—Economic analysis of activated carbon production from spent coffee grounds: Comparative evaluation of different production routes. Energy Convers. Manag. X.

[102] Sridhar A., Ponnuchamy M., Kumar P.S., Kapoor A., Vo D.V.N., Prabhakar S. (2021). Techniques and modeling of polyphenol extraction from food: A review. Environ. Chem. Lett..

[103] Islam M., Malakar S., Rao M.V., Kumar N., Sahu J.K. (2023). Recent advancement in ultrasound-assisted novel technologies for the extraction of bioactive compounds from herbal plants: A review. Food Sci. Biotechnol..

[104] Herzyk F., Piłakowska-Pietras D., Korzeniowska M. (2024). Supercritical Extraction Techniques for Obtaining Biologically Active Substances from a Variety of Plant Byproducts. Foods.

[105] Barik R., Sugunan S., Shafri M.A.B.M. (2024). Pressurized Liquid Extraction for the Isolation of Bioactive Compounds. Bioactive Extraction and Application in Food and Nutraceutical Industries.

[106] García-Roldán A., Piriou L., Jauregi P. (2023). Natural deep eutectic solvents as a green extraction of polyphenols from spent coffee ground with enhanced bioactivities. Front. Plant Sci..

[107] Muniasamy R., Venkatachalam P., Rangarajan V., Samal S., Rathnasamy S. (2023). A comprehensive perspective on sustainable bioprocessing through extractive fermentation: Challenges and prospects. Rev. Environ. Sci. Biotechnol..

[108] Agraso-Otero A., Rebolledo-Leiva R., Entrena-Barbero E., González-García S. (2025). Integrated process design, techno-economic and environmental analysis of chokeberry pomace biorefineries: Phenolic compounds extraction with ethanol or energy production?. Environ. Technol. Innov..

[109] Ingrao C., Platnieks O., Siracusa V., Gaidukova G., Paiano A., Gaidukovs S. (2022). Spent-coffee grounds as a zero-burden material blended with bio-based poly(butylene succinate) for production of bio-composites: Findings from a Life Cycle Assessment application experience. Environ. Impact Assess. Rev..

[110] Rajabi Hamedani S., Colantoni A., Bianchini L., Carnevale M., Paris E., Villarini M., Gallucci F. (2022). Environmental life cycle assessment of spent coffee ground pellet. Energy Rep..

[111] Martinez-Saez N., García A.T., Pérez I.D., Rebollo-Hernanz M., Mesías M., Morales F.J., Martín-Cabrejas M.A., del Castillo M.D. (2017). Use of spent coffee grounds as food ingredient in bakery products. Food Chem..

